# A Comparison of the Perceived Hearing Difficulties of Community and Clinical Samples of Older Adults

**DOI:** 10.1044/2021_JSLHR-20-00728

**Published:** 2021-08-24

**Authors:** Larry E. Humes, Judy R. Dubno

**Affiliations:** a Department of Speech and Hearing Sciences, Indiana University Bloomington; b Department of Otolaryngology - Head & Neck Surgery, Medical University of South Carolina, Charleston

## Abstract

**Purpose:**

This study aimed to compare the perceived hearing difficulties of a community sample of older adults to two clinical samples of older adults, one with no hearing aid experience and the other with hearing aid experience.

**Method:**

Scale scores from the Communication Profile for the Hearing Impaired (CPHI) were analyzed for a community sample of older adults (*N* = 243) and compared to scores from two clinical samples, one without (*N* = 342) and one with prior hearing-aid experience (*N* = 179). General linear model (GLM) analyses were performed to examine the effects of data sample type and other factors on CPHI scale scores. Scores for the Hearing Handicap Inventory for the Elderly (HHIE) were also available for most participants and were analyzed.

**Results:**

GLM analyses of each of the 20 CPHI scale scores showed significant effects of sample type with hearing-loss severity and age most frequently showing significant effects as well. GLM analyses controlling for hearing-loss severity and age across sample types found significant differences on most CPHI scales between the community sample and each of the two clinical samples. Significant differences between the two clinical samples were also found on several CPHI scales and on the HHIE.

**Conclusions:**

Older adults from the community who did not seek help for hearing difficulties self-reported less difficulty and a greater denial or lack of awareness of communication problems than those who sought assistance at an audiology clinic. For those presumed to have sought a hearing evaluation, those acquiring hearing aids perceived greater communication difficulties in all environments, had greater awareness of communication difficulties, were more accepting of their hearing loss, but tended to allocate more responsibility for their difficulties to others, compared to those who sought clinical assistance but did not acquire hearing aids.

The prevalence of hearing loss increases with advancing age (Cruickshanks et al., 2010; Lin et al., 2011). Worldwide, about 20% of persons age 60 years and 50% age 80 years are affected by disabling hearing impairment (Stevens et al. 2013). These prevalence estimates, defined by “disabling” hearing impairment, were based on the pure-tone average for 500, 1000, 2000, and 4000 Hz (PTA4) in the better ear ≥ 35 dB HL. Despite the widespread prevalence of age-related hearing loss according to this definition, industry surveys and epidemiological studies have consistently documented that only about 15%–30% of adults with mild-to-moderate hearing loss obtain hearing aids (Bisgaard & Ruf, 2017; Chia et al., 2007; Chien & Lin, 2012; Dalton et al., 2003; Dawes et al., 2014; Kochkin, 1993a, 1993b, 1993c, 2000, 2009; Popelka et al., 1998; Sangster et al., 1991; Smeeth et al., 2002; Ward et al., 1993; Wilson et al., 2010).

Due to the poor uptake of hearing aids, millions of older adults with untreated hearing loss continue to suffer broader consequences. It is well known, for example, that the loss of audibility alone causes many difficulties for everyday speech communication, including poor speech perception (e.g., Humes & Dubno, 2010) and increased listening effort (e.g., Pichora-Fuller et al., 2016), among others. Furthermore, untreated hearing loss can lead to a variety of psychosocial problems, including social isolation, with most studies focusing on depression (e.g., Cosh et al., 2019). There is mounting evidence, moreover, that untreated hearing loss can have a negative impact on cognitive function and that hearing aids may help to reduce that impact (e.g., Amieva & Ouvrard, 2020). In summary, the poor uptake of hearing aids by older adults with mild-to-moderate hearing loss is a serious problem with broad potential consequences on everyday function and well-being.

An assumption underlying the foregoing is that the audiogram (and better-ear PTA4 derived from it) is the most appropriate way to define candidacy for amplification as well as the potential benefit to be experienced from hearing aids. It has long been known that older adults with identical audiograms can have markedly different perceived hearing difficulties (e.g., Ventry & Weinstein, 1982, 1983; Weinstein & Ventry, 1983). If one assumes that the primary factor leading to a visit to an audiology clinic for a hearing evaluation would be perceived hearing loss but hearing-aid candidacy is determined audiometrically, then analyses by Edwards (2020) suggest that nearly two thirds of those with perceived hearing difficulty have no measurable audiometric hearing loss and therefore would not be defined audiometrically as a hearing-aid candidate. It has been argued previously that self-reported hearing difficulty is superior to impairment definitions based on the pure-tone threshold (see review by Vermiglio et al., 2018) Furthermore, perceived hearing difficulties have repeatedly been identified as the key factor for hearing-aid uptake and use (e.g., Hickson et al., 2014; Knudsen et al., 2010; Laplante-Lévesque et al., 2012; Pronk et al., 2017; Ratanjee-Vanmali et al., 2019; Sawyer et al., 2019; Simpson et al., 2019).

Among those who do seek help, many do not follow through with recommendations to obtain hearing aids. For example, Yueh et al. (2010) screened the hearing of 1,382 older adults, about 48% of whom failed the screen. Of those who failed, about 46% followed up with an audiologist to schedule a full hearing evaluation and subsequently kept that appointment. Of those who kept their appointments for detailed hearing evaluations, about 75% were considered hearing aid candidates by their audiologists based on the audiogram, but only about one third of candidates were fitted with hearing aids and were using them a year later. Thus, many who do have their hearing evaluated at an audiology clinic and are informed that they are candidates for hearing aids choose not to follow-up on those recommendations. Although many factors may impact the older adult's decision to pursue amplification following audiometric evaluation, self-perceived hearing difficulties and their reactions to them may be paramount. If audiometrically defined hearing loss was the criterion for hearing-aid candidacy, as many as 25 million of 37 million adults in the United States with perceived hearing difficulties would not be considered candidates for hearing aids (Edwards, 2020), an observation consistent with earlier analyses by Martin and Champlin (2000).

Perhaps the most detailed assessment of perceived hearing difficulties, and the individual's reaction to those difficulties, is the Communication Profile for the Hearing Impaired (CPHI; Demorest & Erdman, 1986, 1987). The full CPHI includes 163 items and 25 scales, which comprise five subsets of scales organized around common themes of Communication Performance (CP), Communication Importance (CI), Communication Environment (CE), Communication Strategies (CS), and Personal Adjustment (PA) to impaired hearing. Generally, the key factors that influence scores on many of the CPHI scales among older adults are the severity of hearing loss, age, and education level, with mixed findings regarding the influence of sex (Erdman & Demorest, 1998b; Garstecki & Erler, 1999).

Two studies have examined differences in CPHI scores between older adults seen at the audiology clinic who were advised to pursue hearing aids and did (adherents) and those who received the same advice but chose not to do so (non adherents). Garstecki and Erler (1998) obtained CPHI scores from 60 older adults (35 females) who were adherents and 71 older adults (33 females) who were nonadherents. Humes et al. (2003) examined differences in CPHI scores among three groups of older adults who were matched for average audiograms, age, and sex. Performance of one group of nonadherents (*n* = 26) was compared to two groups of adherents, one group who had accepted hearing aids after the 30-day trial period and was still using them 6 months later (*n* = 26) and one group who had discontinued using their hearing aids six months later (*n* = 24), about half of whom returned their hearing aids during the initial 30-day trial. Significant differences in CPHI scale scores were observed on several CPHI scales between adherents and nonadherents in the studies by Garstecki and Erler (1998) and Humes et al. (2003).

Aside from these two studies of CPHI scores in adherents and nonadherents, there are several large studies of CPHI scores from clinical samples. The CPHI was originally normed, for example, on a large sample of 827 patients at the Walter Reed Army Medical Center (Demorest & Erdman, 1987). Garstecki and Erler (1999) extended application of the CPHI to older adults by assessing 301 adults between the age of 65 and 93 years, 47% of whom were female. The data set developed by Erdman and Demorest (1998a) represented an effort to develop a “clinical norm” for the CPHI that would be more representative of clinical samples than the original samples from Walter Reed Army Medical Center (Demorest & Erdman, 1987). Although the ages across the five clinics included in the analyses ranged from 16 to 97 years, the mean ages across the clinics ranged from 63.1 to 66.1 years. Erdman and Demorest (1998a) observed that the pattern of mean scale scores across CPHI scales was very similar for all data sets obtained previously, by these authors and others, with differences primarily confined to overall adjustment of the pattern by a constant amount. In summary, when the data from hundreds of older adults who have visited a clinic for an audiology evaluation are pooled, a detailed profile emerges from the CPHI describing their communication difficulties and their reactions to them.

It appears, however, that little is known about the communication difficulties and reactions to them for older adults in the broader community, that is, those who have not sought help at an audiology clinic or acquired hearing aids. To our knowledge, detailed analyses of the communication problems experienced and the reactions to those problems via the CPHI have not been provided for such a community-based sample. Rather, to date, the focus of the CPHI has been to obtain profiles of communication difficulties and reactions to those difficulties from individuals, including older adults, who have been seen at an audiology clinic. The CPHI was envisioned as a tool to assess perceived hearing difficulties and reactions to those problems and guide the subsequent rehabilitation of adults with or without measurable hearing loss as determined at the audiologic evaluation.

This study sought to fill this gap by analyzing CPHI scores from 764 older adults divided into three groups: (a) a convenience sample of the Charleston, South Carolina community recruited for participation in a longitudinal study of hearing over the adult lifespan at the Medical University of South Carolina (MUSC; *n* = 243); (b) a combined clinical sample comprised of those referred to the MUSC longitudinal study after being seen at the MUSC audiology clinic who were not also current hearing aid wearers (*n* = 77), and older adults from the Bloomington, Indiana community who had responded to ads for participation in hearing-aid research at Indiana University (IU) and had not worn hearing aids previously (*n* = 265), resulting in a total population size of 342 for this group; and (c) a combined MUSC and IU clinical sample who were either current hearing aid wearers (MUSC; *n* = 52) or had ever worn hearing aids (IU; *n* = 127) for a total population size of 179 for this group of older adults. As will be seen, the nonclinical MUSC community sample of older adults includes many older adults with impaired hearing, yet they have opted, it appears, to not pursue audiologic evaluation, albeit they did volunteer to participate in a longitudinal study of hearing. Also, it is not possible to confirm from the information available whether any of these individuals sought hearing evaluations outside of the MUSC clinic. It is assumed here that the vast majority did not do so, consistent with the modeling of Edwards (2020), who considered this group to represent approximately two thirds of those with known hearing loss. Specifically, of the 372 individuals in the MUSC data set, 243 or 65.3% had not been seen in the MUSC audiology clinic or were not currently using hearing aids. This is in very good agreement with the modeling of Edwards (2020). Finally, we relied on self-report to establish whether an individual was a current hearing aid user or had ever used hearing aids.

In the pages to follow, we examine whether there are self-reported differences in communication difficulties and reactions to them among older adults in the community who did not seek an audiology evaluation or acquire hearing aids (Community sample), those who obtained an audiology evaluation but did not acquire hearing aids (Clinic–No Hearing Aid [Clinic–No HA] sample), and those who obtained an audiology evaluation and acquired hearing aids (Clinic–Hearing Aid [Clinic–HA] sample). Through such evaluation, it is hoped to gain a better understanding of whether and how differences in perceived hearing difficulties underlie the help-seeking behaviors of older adults with impaired hearing.

## Method

### Participants

There were three samples of older adults included in these analyses. The Community sample was entirely from the Charleston, SC area and responded to ads placed in the community for participation in a longitudinal study of hearing over the adult lifespan. Of the 243 members of this sample, 141 (58%) were female. Ages for this sample ranged from 50 to 88 years with a mean age of 68.6 years (*SD* = 6.1 years) for the females and a mean age of 68.2 years (*SD* = 6.5 years) for the males. The Clinic–No HA sample included individuals from the Charleston, SC area recruited for the longitudinal study of hearing (*n* = 77) and individuals from the Bloomington, IN area recruited for a study of hearing-aid outcomes (*n* = 265). All individuals in this combined sample (*n* = 342) were recruited from the affiliated university clinics or were volunteering for a study on hearing-aid outcomes in which they knew hearing-aid purchase was required. At the time of the completion of the CPHI, however, no individuals in this group self-reported current hearing aid use (MUSC data set) or ever having used hearing aids (IU data set). Of the 342 individuals in this sample, 39% were female. Ages ranged from 57 to 89 years with a mean age of 74.5 years (*SD* = 7.0 years) for the females and 70.8 years (*SD* = 6.8 years) for the males. The Clinic–HA sample included 127 members of the Bloomington, IN community who had volunteered for the study of hearing-aid outcomes who reported that they had previously worn hearing aids and 52 members of the Charleston, SC community who had volunteered for the longitudinal study of hearing who reported that they were currently using hearing aids. As noted previously, about two thirds of those from the Bloomington community who had reported prior hearing aid use were current hearing aid users. The Clinic–Hearing Aid sample, total *n* = 179, ranged in age from 52 to 87 years with a mean age of 75.7 years (*SD* = 6.7 years) for the females and a mean age of 71.8 years (*SD* = 7.0 years) for the males.

For the IU data set, the following inclusion criteria applied: (a) age between 60 and 89 years; (b) hearing loss that was flat or gently sloping (from 250 to 4000 Hz, no inter-octave change in hearing thresholds of more than 20 dB); (c) hearing loss that was of sensorineural origin (normal tympanometry and air–bone gaps no greater than 10 dB at three or more frequencies); (d) hearing loss that was bilaterally symmetrical (interaural difference within 30 dB at all octave and half-octave intervals from 250 to 4000 Hz); (e) pure-tone thresholds within the following ranges at frequencies of 250, 500, 1000, 1500, 2000, 3000, 4000, and 6000 Hz, respectively: 5–85, 5–85, 10–90, 20–95, 25–95, 30–120, 30–120, and 30–120 dB HL (ANSI, 1996); (f) no known medical or surgically treatable ear-related condition; (g) no known fluctuating or rapidly progressing hearing loss; (h) no cognitive, medical, or language-based conditions that may have limited the participant's ability to complete the procedures used in the longitudinal study of outcome measures; (i) no use of medications that could affect hearing or cognition; and (j) completion of a signed medical clearance form, or waiver of such by the participant, and a signed informed consent form. The consent form and all procedures used in this study were approved by the IU Bloomington Institutional Review Board.

For the IU data set, from 1997 to 2008, there was a total of 930 individuals who responded to the ads and were screened for study eligibility. For the period 2004–2008, when this information was collected more formally and in detail, approximately 30% of those screened were found to be ineligible. This percentage was applied to the entire sample of 930 screened individuals, leaving 651 eligible individuals. Of the estimated 651 eligible individuals, 392 (60.2%) enrolled in the studies of hearing-aid outcomes. They paid the full purchase price for the devices at the time of enrollment and were paid as research subjects for the completion of other data-collection sessions.

As noted above, an audiogram was completed as a part of the eligibility criteria for the IU data set. After completion of the audiological evaluation, the results were explained briefly to the individual and, if eligible for study enrollment, they were informed about the study at this time. As noted, the study was investigating hearing-aid outcomes and they were informed that they would be required to purchase hearing aids for that study. Those interested in participating were consented and enrolled. No other counseling was provided to these individuals until after the hearing-aid fitting, which took place after the completion of all pre-fit measures, including the CPHI.

For the MUSC data set, the following inclusion criteria applied: (a) age 18 and over, (b) good general health (well enough to attend several lab visits), (c) no evidence of conductive hearing loss or active otologic/neurologic disease, and (d) ability to provide reliable results for a majority of the protocol measures. The consent form and all procedures used in this study were approved by the MUSC Institutional Review Board. During a screening visit, the study was explained briefly to the individual and, if eligible for study enrollment, they were informed about the study. Those interested in participating were consented and enrolled. No counseling was provided to study participants until after completion of the CPHI or Hearing Handicap Inventory for the Elderly (HHIE).

For the MUSC data set, from the start of this longitudinal study in 1987, approximately 90% of individuals screened for eligibility were enrolled in the study. The average attrition rate is approximately 14%; however, a large proportion of participants who withdrew from the study were less than 50 years of age, which is the minimum age of participants whose baseline data were included in the current analyses.

### Procedure

Prior to completing the CPHI, each participant in each group received a complete audiological evaluation at study enrollment, including pure-tone and speech audiometry, as well as immittance measures. The CPHI was administered at each site using a paper-and-pencil response format with the participant seated in a quiet room. Booklets were provided to the participant that contained the standardized written instructions for each section of the CPHI. For each item, one of five response choices is selected. Computer software supplied by Marilyn Demorest was used to score the CPHI (Demorest & Erdman, 1989a). As indicated by Demorest and Erdman (1989b), the three CI scales of the CPHI are considered extra or optional measures and not a central part of the CPHI, which reduces the CPHI to 145 items and 22 scales. Furthermore, two CP scales, one for average conditions and one for adverse conditions, are a recalculation of the responses forming three other CP scales and are excluded due to the redundancy (Demorest & Erdman, 1989b). This leaves 20 of the 25 CPHI scale scores as the core CPHI measures, which were the scales analyzed here.

In addition, almost 80% of the participants included in the current CPHI analyses had also completed the full 25-item HHIE (Ventry & Weinstein, 1982). The HHIE results will be examined as a means of validating the findings observed for the CPHI.

### Preliminary Data Analyses

Chi-squared analysis indicated that there were significant differences in the proportion of males and females across the three samples, χ^2^ (2) = 45.5, *p* < .001, and follow-up *z*-tests of the proportion of females in each group found that all three samples differed from one another (*p* < .05). The Community sample was 58.0% female, the Clinic–No HA sample was 38.6% female, and the Clinic–HA sample was 26.3% female. Because of these significant differences in male/female proportions for each sample, as well as the mixed findings regarding the influence of this variable on CPHI scores as noted earlier, sex will serve as another independent variable for all comparisons of the three samples.

When the age composition of each of the three samples was analyzed using general linear models (GLMs) with sex as another independent variable, significant main effects of sex, *F*(1, 758) = 24.3, *p* < .001, and sample type, *F*(2, 758) = 38.6, *p* < .001, emerged. The interaction between sex and sample type, however, was also significant, *F*(2, 758) = 4.7, *p* < .01. Post hoc paired comparisons revealed that there were no significant differences in age between the males and females for the Community sample (*p* > .05), but females were significantly older than males (*p* < .05) for the other two samples. As a result, as well as the prior literature reviewed earlier that documented effects of age on CPHI scores, age will also serve as a covariate when comparing CPHI results across sample types.

**Figure 1.**
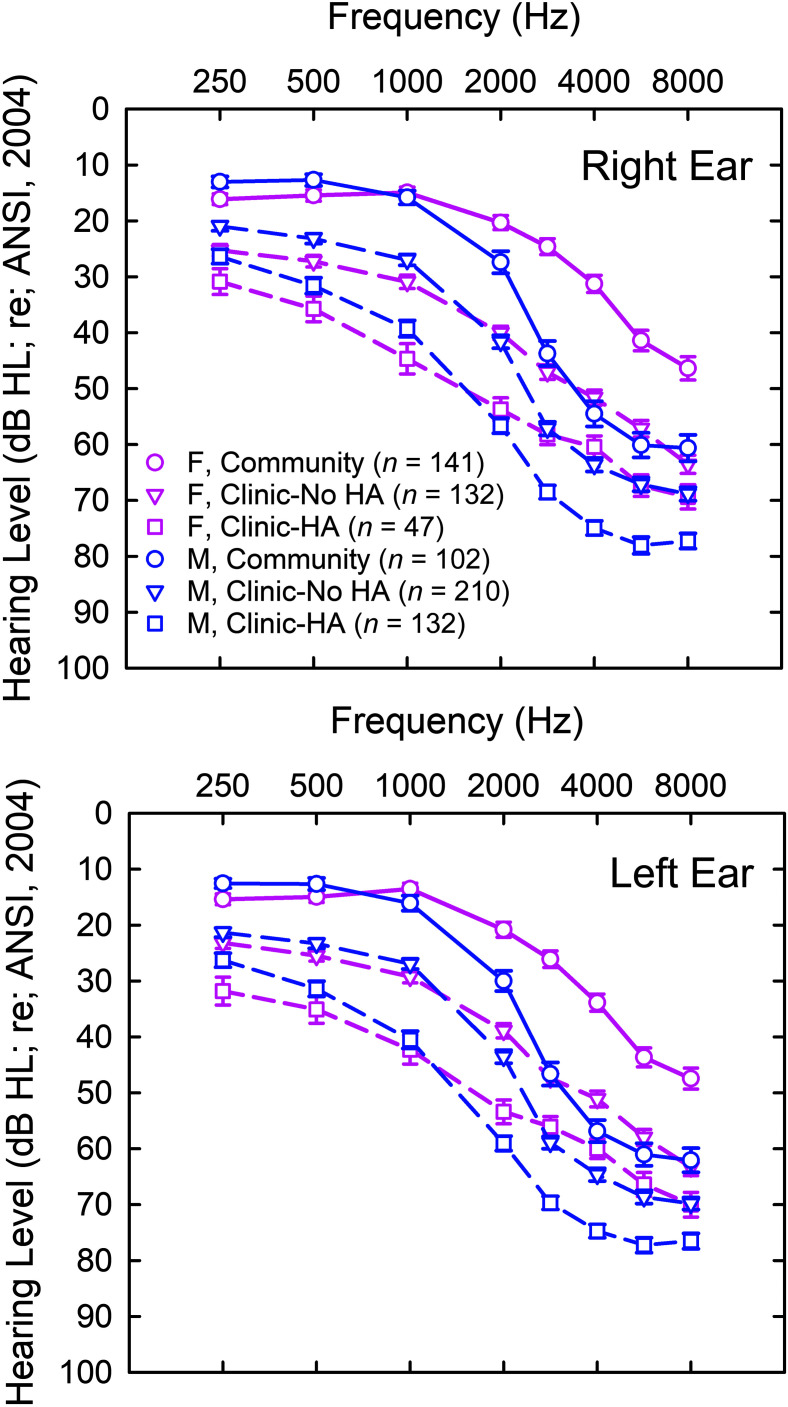
Means and standard errors for each of the three sample types, Community, Clinic-No Hearing Aid (HA), Clinic-HA, for females (pink) and males (blue). Top panel shows the data for the right ear and bottom panel for the left ear. ANSI = American National Standards Institute.

The three data sample types also differed regarding their pure-tone air-conduction hearing thresholds. Mean hearing thresholds for the males and females comprising each sample type are shown in Figure 1 with the data for the right ear in the top panel and for the left ear in the bottom panel. The patterns visible in Figure 1 are similar for both ears. Generally, the severity of hearing loss increases as the sample type changes from Community to Clinic–No HA to Clinic–HA. In addition, males generally have poorer hearing than females within each sample type, but this is mainly confined to frequencies at and above 2000 Hz. The patterns of hearing loss visible in Figure 1 were confirmed statistically using GLM analyses with between-subject factors of sex and sample type and a within-subject factor of test frequency. For the right ear, significant main effects were observed for sample type, *F*(2, 736) = 175.8, *p* < .001, and sex, *F*(1, 736) = 32.0, *p* < .001, but the interaction of sample type and sex was also significant, *F*(2, 736) = 5.9, *p* < .01. Regarding the effects of test frequency for the right ear, the main effect was significant, *F*(7, 5152) = 1,253.9, *p* < .001, as were all of the interactions with frequency: Frequency × Sample Type, *F*(14, 5152) = 11.3, *p* < .001; Frequency × Sex, *F*(7, 5152) = 77.4, *p* < .001; Frequency × Sample Type × Sex, *F*(14, 5152) = 2.0, *p* < .05. For the left ear, significant main effects were observed for sample type, *F*(2, 738) = 170.9, *p* < .001, and sex, *F*(1, 738) = 51.0, *p* < .001, but the interaction of sample type and sex was also significant, *F*(2, 738) = 5.0, *p* < .01. Regarding the effects of test frequency for the right ear, the main effect was significant, *F*(7, 5166) = 1,441.6, *p* < .001, as were all of the interactions with frequency: Frequency × Sample Type, *F*(14, 5166) = 9.7, *p* < .001; Frequency × Sex, *F*(7, 5166) = 75.1, *p* < .001; Frequency × Sample Type × Sex, *F*(14, 5166) = 2.2, *p* < .01. When these GLM analyses of the audiograms in Figure 1 were performed again with age as a covariate, all the significant effects were retained except for the interaction of sample type and sex in the left ear, *F*(2, 737) = 2.5, *p* > .05.

Difference in audiograms between males and females, especially in the higher frequencies, are well known and are not of primary interest here other than to note that examination of the effects of sex on CPHI scale scores must control for differences in hearing loss between males and females. The differences in audiograms among the three sample types, moreover, are also expected. Garstecki and Erler (1998) found differences in hearing loss between adherents, similar to the Clinic–HA sample here, and nonadherents, similar to the Clinic–No HA sample in these analyses. Clearly, differences in the amount of hearing loss may underlie the motivation to go to the audiology clinic for help or to pursue amplification after doing so. Even the magnitude of hearing loss as indicated by the audiograms of the Community sample, however, suggest that these individuals could benefit from a hearing evaluation and the use of hearing aids, yet it appears that they chose to do neither, perhaps because of differences in perceived hearing difficulties independent of hearing loss. In our analyses of differences in CPHI scale scores among the three sample types to follow, both age and better-ear four-frequency (500, 1000, 2000, and 4000 Hz) pure-tone average, referred to here as PTA4, will serve as covariates. We chose the better-ear PTA4 over right ear, left ear, or worse ear because this factor is more likely a key audiometric contributor to the individual's performance in many everyday situations assessed by the CPHI. If no significant differences in CPHI scores among the sample types are observed when controlling for differences in age and hearing loss, then it would be difficult to argue that their help-seeking behavior was driven by their perceived hearing difficulties and their reactions to them.

## Results

**Figure 2.**
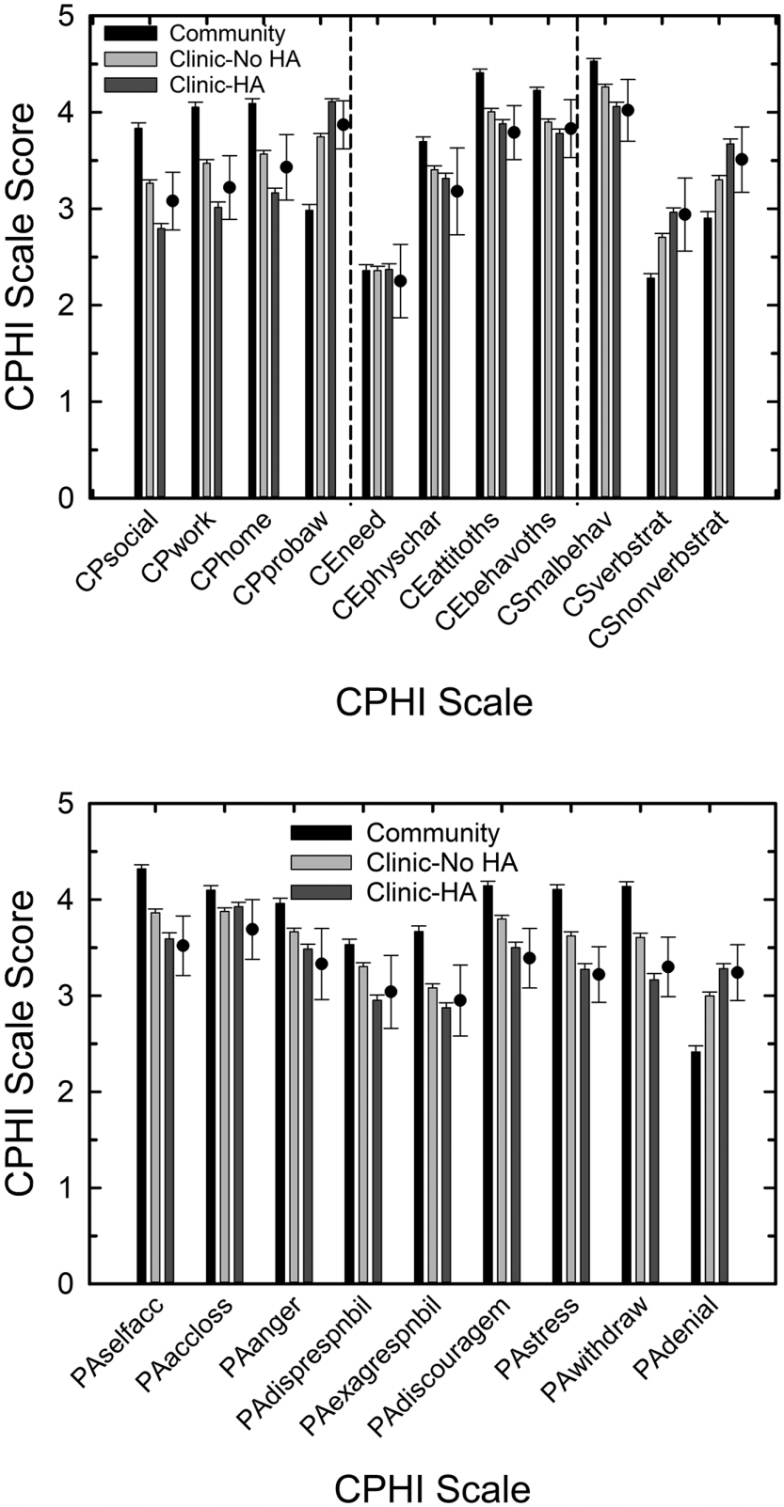
Means and standard errors from the three sample types (Community, Clinic–No Hearing Aid [Clinic–No HA], Clinic–Hearing Aid [Clinic–HA]) included in these analyses. Bottom panel shows the results for the nine Personal Adjustment (PA) scales of the Communication Profile for the Hearing Impaired (CPHI) and the top panel shows the results for the other 11 CPHI scales (CP = Communication Performance; CE = Communication Environment; CS = Communication Strategies). The filled circles with errors bars adjacent to each set of three vertical bars represent the means and standard deviations for these same CPHI scales for a very similar clinical data set (*N* = 1,004; Erdman & Demorest, 1998a).

The vertical bars in Figure 2 present the means and the standard errors for the 20 CPHI scales for each of the three sample types: Community, Clinic–No HA, and Clinic–HA. The top panel displays the scales for CP scales, the CE scales, and the CS scales of the CPHI; whereas, the bottom panel presents the results for the PA scales of the CPHI. The CPHI scale scores are calculated such that higher scores are “better”; better communication performance, more favorable communication environment, more frequent use of communication strategies, and better personal adjustment to impaired hearing.

The filled circles with error bars appearing in both panels of Figure 2 are the means and standard deviations for the clinical norms (*N* = 1,004) derived across five sites by Erdman and Demorest (1998a). Scale names and the abbreviations used in Figures 2 [Fig F3] [Fig F4] through 5 are provided in Table 1. Recall that nearly 40% of those included in these clinical norms were hearing-aid wearers. Across all 20 scales, good agreement, within 1 *SD* of the normative mean, is observed between the norms and the means of the Clinic–HA sample with similarly good agreement between the norms and the means of the Clinic–No HA sample for 18 of the 20 scales. The Clinic–No HA sample shows means slightly higher than the clinic norms for the PA-stress and PA-withdrawal CPHI scales. In general, however, the clinical data reported here are in good agreement with the previously published clinical norms for the CPHI (Erdman & Demorest, 1998a).

**Table 1.** CPHI scale names from Demorest and Erdman (1986, 1987) and the abbreviations used for those scales in Figures 2 [Fig F3] [Fig F4] through 5.

**Scale name**	**Scale abbreviation**
Communication Performance–Social situations	CPsocial
Communication Performance–At Work	CPwork
Communication Performance–At Home	CPhome
Communication Performance–Problem Awareness	CPprobaw
Communication Environment–Communication Need	CEneed
Communication Environment–Physical Characteristics	CEphyschar
Communication Environment–Attitudes of Others	CEattitoths
Communication Environment–Behaviors of Others	CEbehavoths
Communication Strategies–Maladaptive Behaviors	CSmalbehav
Communication Strategies–Verbal Strategies	CSverbstrat
Communication Strategies– Nonverbal Strategies	CSnonverbstrat
Personal Adjustment–Self–Acceptance	PAselfacc
Personal Adjustment–Acceptance of Loss	PAaccloss
Personal Adjustment–Anger	PAanger
Personal Adjustment–Displacement of Responsibility	PAdisprespnbil
Personal Adjustment–Exaggeration of Responsibility	PAexagrespnbil
Personal Adjustment–Discouragement	PAdiscourage(m)
Personal Adjustment–Stress	PAstress
Personal Adjustment–Withdrawal	PAwithdraw
Personal Adjustment–Denial	PAdenial

Visual inspection of the means in Figure 2 suggests that the means for the Community sample are typically higher (indicating better communication performance and personal adjustment) than one or both of the means for the clinic samples with the likely exception of the CE-need scale (top panel). To evaluate this further, a one-way analysis of variance, with sample type as the only independent variable and no covariates, was performed for each of the 20 CPHI scales. Except for the CE-need CPHI scale, a significant effect (*p* < .001) of sample type was found, all *F*(2, 765) > 7.23. Moreover, the partial eta-squared effect sizes for the 19 significant results were large in 11 cases, medium in seven, and small for one (PA-accept loss; Cohen, 1988). Post hoc pairwise comparisons at a Bonferroni-adjusted significance level of *p* < .05, that is, *p* < (.05/19) or .0026, revealed that, for 15 of the 19 significant effects of sample type, all three samples differed from one another. For the remaining four significant effects of sample type (CE-physical characteristics, CE-attitudes of others, CE-behavior of others, and PA-accept loss), the Community sample had higher scale scores than both of the two clinic samples (indicating better outcomes) and the two clinics samples did not differ from one another. Where significant effects of sample type were observed, that is, 19 of the 20 CPHI scales, the Community sample had significantly higher scores for 15 scales, but had significantly lower scores for four CPHI scales, CP-problem awareness, CS-verbal strategies, CS-nonverbal strategies, and the PA-denial scales.

As noted previously, the sample types differed significantly in hearing loss, age, and sex. The significant effects of sample type on CPHI scales appearing in Figure 2 could be due to these mediating factors. To examine this, GLM analyses were again performed for each of the 20 CPHI scales. The two independent variables in each analysis were sex and sample type with better-ear PTA4 and age as covariates in each analysis. Regarding the PTA4 covariate, this had a significant effect on 19 of 20 CPHI scale scores, all *F*(1, 755) > 12.2, *p* < .001, except for CE-physical characteristics, *F*(1, 755) = 3.3, *p* > .05. Moreover, the partial eta-squared effect sizes for better-ear PTA4 were large for five scales (all four CP scales and PA-withdrawal), small for two (CE-communication need, PA-accept loss), and medium for all other CPHI scales (Cohen, 1988). Regarding the age covariate, age had a significant effect on seven of the 20 CPHI scales, *F*(1, 755) > 4.2, *p* < .05. Based on partial eta-squared effect sizes for age, six of the seven had small effect sizes (CP-work, CP-problem awareness, CE-physical characteristics, CE-attitudes of others, CS-verbal strategies, CS-nonverbal strategies) and one had a medium effect size (CE-communication need).

**Figure 3. F3:**
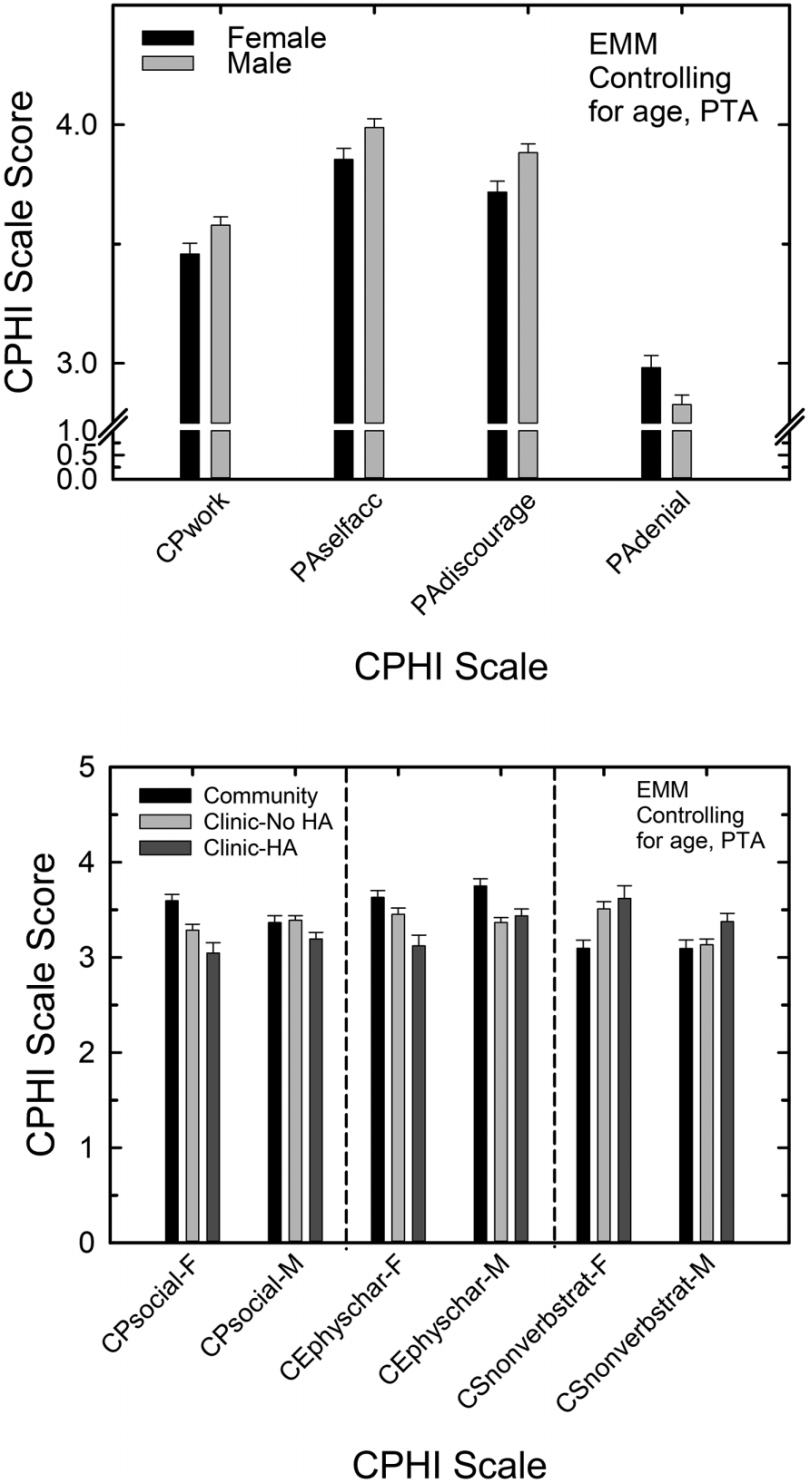
Significant main effects (top) and interactions (bottom) with sex are shown for the Communication Profile for the Hearing Impaired (CPHI) scales having such effects. Estimated marginal means (EMMs) and standard errors are shown with age and better-ear four-frequency pure-tone average (PTA4) as covariates. F = female; M = male.

Regarding the effect of sex on CPHI scale scores, a significant main effect, without interaction with sample type, was observed for four CPHI scales, all *F*(1, 755) > 4.3, *p* < .05. The estimated marginal means, at a mean PTA4 of 34.0 dB HL and a mean age of 71.1 years, are shown in the top panel of Figure 3 for the four CPHI scales with significant main effects of sex. In three of the four cases, males had significantly higher CPHI scale scores and, for one (PA-denial), a significantly lower scale score than females. The partial eta-squared effect sizes of the main effect of gender were small for all four CPHI scales (Cohen, 1988).

For three additional CPHI scales, CP-social, CE-physical characteristics, and CS-nonverbal strategies, there were significant interactions between sex and the other independent variable, sample type, all *F*(2, 755) > 3.1, *p* < .05. The partial eta-squared effect sizes for these three significant interactions between sex and sample type were all small (Cohen, 1988). The bottom panel of Figure 3 shows the significant interactions between sex and sample type. The interactions are apparent visually in these data. Confirmation of the apparent visual trends via follow-up Bonferroni-adjusted paired comparisons showed that, for the CP-social scale (left panel), for females, all three sample types differed significantly (*p* < .05) from one another and in the direction reflected in the bottom panel of Figure 3. In contrast, for males, only the Community and Clinic–HA samples differed significantly (*p* < .05). For the CE-physical characteristics scale (middle panel), for females, all three sample types again differed significantly (*p* < .05) from one another. For males, the Community sample differed significantly (*p* < .05) from both clinic samples but the two clinic samples did not differ significantly (*p* > .05) from one another. Finally, for the CS-nonverbal strategies CPHI scale, as shown in the bottom panel of Figure 3, for females, the Community sample had significantly lower scale scores than each of the clinic samples, but the clinic samples did not differ significantly from one another. In contrast, for males, the Clinic–HA sample had a significantly (*p* < .05) higher scale score than each of the other two sample types, but the Community and Clinic–No HA samples did not differ significantly (*p* > .05) from one another on this scale.

**Figure 4. F4:**
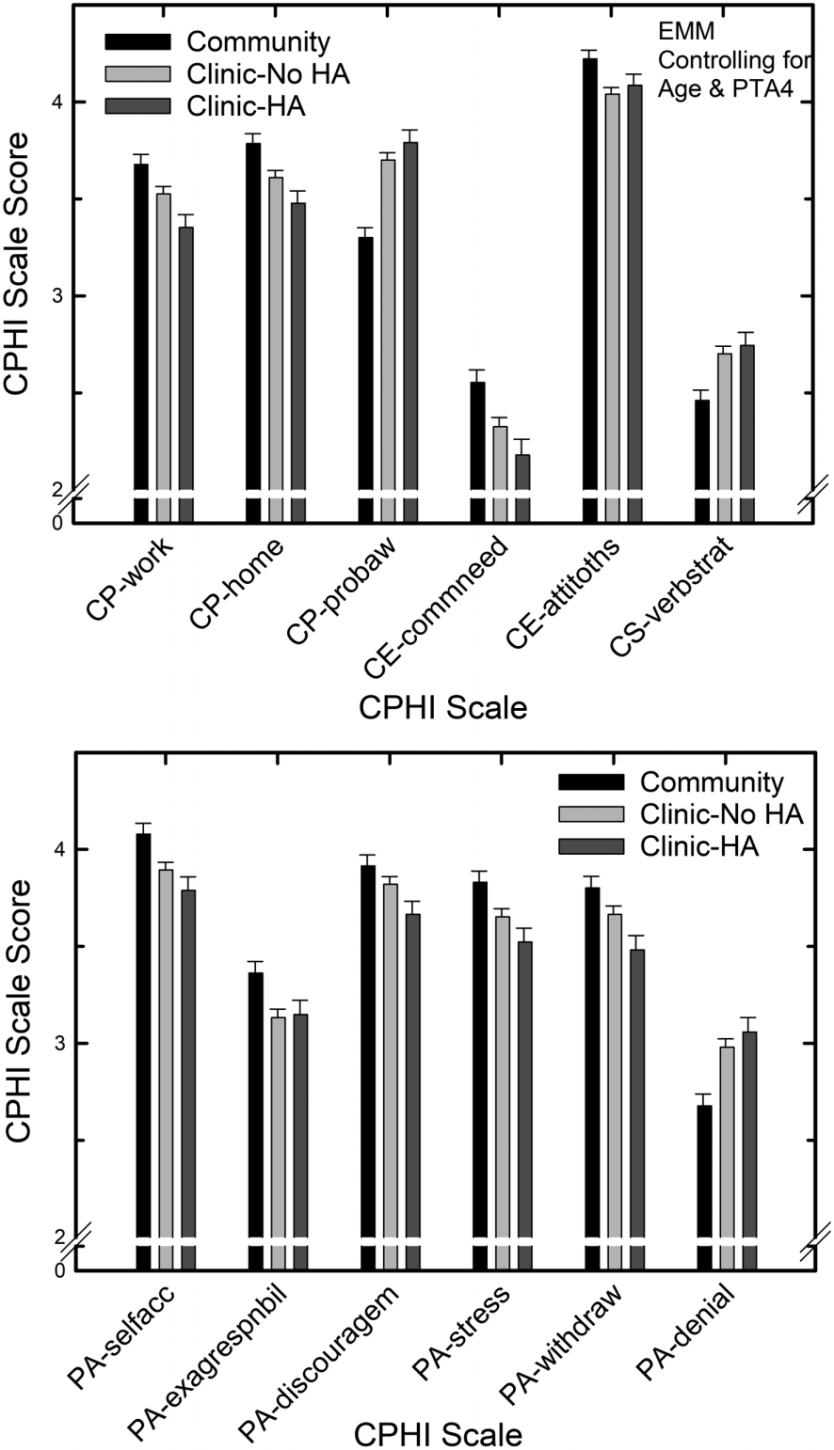
Estimated marginal means (EMMs) and standard errors for each of the Communication Profile for the Hearing Impaired (CPHI) scales showing significant effects of sample type for the general linear model analysis with age and better-ear four-frequency pure-tone average (PTA4) as covariates. Clinic–No HA = Clinic–No Hearing Aid; Clinic–HA = Clinic–Hearing Aid.

Finally, the main effect of sample type, free from significant interactions with sex, was significant for 12 of the 20 scales after controlling for age and better-ear PTA4, all *F*(2,755) > 3.3, *p* < .05, with 11 of the 12 having small partial eta-squared effect sizes and one (CP-problem awareness) having a medium effect size (Cohen, 1988). Figure 4 shows the estimated marginal means and standard errors for the three sample types, controlling for age and better-ear PTA4, for the 12 CPHI scales with significant effects of sample type. The mean age and PTA4 values for these estimated marginal means were 71.1 years and 34.0 dB HL, respectively. The top panel shows the six CP, CE, and CS scales and the bottom panel shows the six PA scales with significant main effects of sample type. In four of the six cases in the top panel of Figure 4, the Community sample type was significantly different (*p* < .05) from the two clinic sample types but the latter did not differ from one another. The two exceptions were CP-work, for which the Community sample only differed significantly (*p* < .05) from the Clinic–HA sample, and CE-attitudes of others, for which the Community sample differed only from the Clinic–No HA sample. Regarding the six PA scales in the bottom panel of Figure 4, for three scales (PA-self accept, PA-stress, and PA-denial), the Community sample differed significantly (*p* < .05) from the two clinic samples but the two clinic samples did not differ from one another. For two PA scales, PA-discouragement and PA-withdrawal, the only significant paired comparison was between the Community and Clinic–HA samples. For the remaining PA scale in the bottom panel of Figure 4, PA-exaggerated responsibility, only the Community and the Clinic–No HA sample types differed significantly (*p* < .05).

In summary, these GLM analyses found significant main effects of sample type, after controlling for sample differences in age and better-ear PTA4, on 12 of the 20 CPHI scales and the prevailing pattern was for the Community sample to differ significantly from one or both of the clinic samples with those clinic samples not differing from one another. In addition, recall that there were three other CPHI scales for which there were significant interactions between sample type and sex (CP-social, CE-physical characteristics, and PA-nonverbal strategies). When the effects of sample type were examined separately for each sex for these three CPHI scales, the Community sample always differed from one or both clinic samples. In total, the Community sample differed from the clinic samples on 15 of the 20 CPHI scales after controlling for age and better-ear PTA4. The direction of the difference was not consistent across the CPHI scales such that sometimes the Community sample had higher scale scores and for other scales that sample had the lowest score.

**Figure 5.**
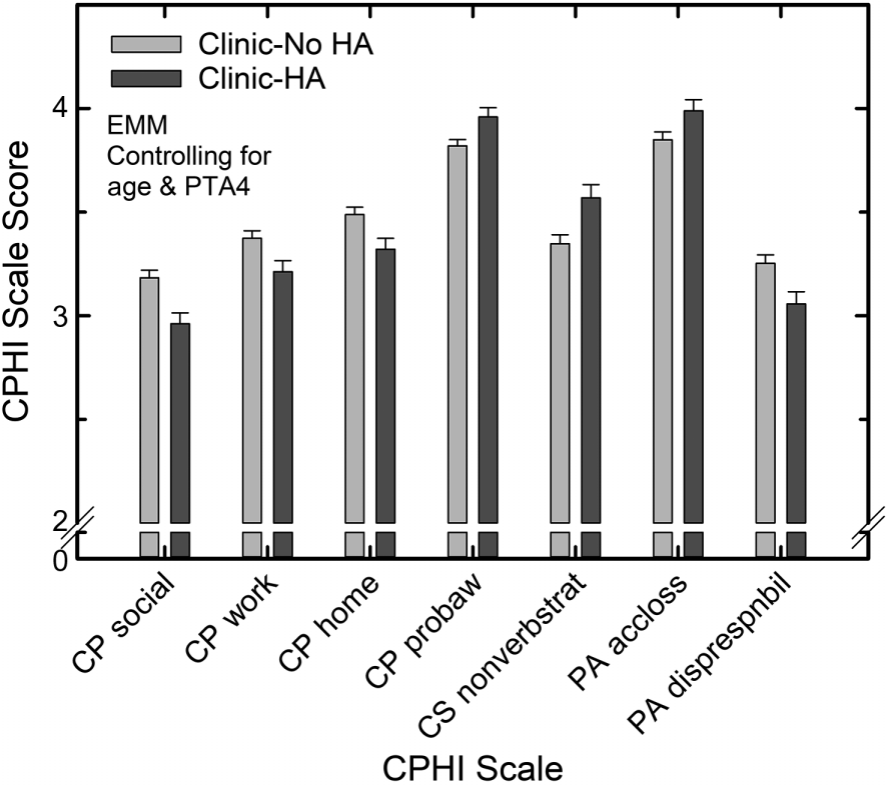
Estimated marginal means (EMMs) and standard errors for each of the CPHI scales showing significant differences between the two clinical samples (No Hearing Aid [Clinic–No HA] and Hearing Aid [Clinic–HA]) from the general linear model analysis with age and better-ear four-frequency pure-tone average (PTA4) as covariates.

For the most part, however, differences were seldom observed between the two clinic samples, those with and without hearing aids. To examine this more closely and with greater statistical power, another series of GLM analyses was performed comparing only the two clinical samples and removing sex as a second independent variable. As noted earlier, sex was found to have a significant effect somewhat infrequently and these effects were not the primary focus of this evaluation. Age and better-ear PTA4 remained as covariates. PTA4 was a significant covariate for 19 of the 20 CPHI scales, all *F*(1, 516) > 8.1, *p* < .01, and mostly medium or large effect sizes (partial eta-squared > .035) were observed. Age was a significant covariate for six of 20 CPHI scales, all *F*(1, 516) > 4.0, *p* < .05. When statistically controlling for better-ear PTA4 and age, significant differences were observed between the Clinic–No HA and the Clinic–HA samples on seven of the 20 CPHI scales, all *F*(1, 516) > 4.1, *p* < .05. Partial eta-squared effect sizes were small for five of the six scales and medium for the remaining scales with significant effects of sample type. Figure 5 provides the estimated margin means and standard errors at a mean age of 72.4 years and a mean better-ear PTA4 of 39.9 dB HL for the seven CPHI scales with significant differences between the two clinical samples. The general pattern that emerges is that, while controlling for age and hearing loss, older adults who acquired hearing aids perceived their communication performance to be poorer than those who did not acquire hearing aids (CP-social, CP-work, CP-home). In addition, adherents were more aware of communication problems (CP-problem awareness), made more frequent use of nonverbal strategies to facilitate communication (CS-nonverbal), were more accepting of their hearing loss (PA-accept loss) and were less likely to place the responsibility for communication difficulties elsewhere (PA-displaced responsibility) than nonadherents.

### Validation with the HHIE

In addition to the full CPHI, 596 of the 764 (78%) participants included in the MUSC and IU data sets also had completed the full 25-item HHIE measure. Those with HHIE scores available constituted 100% of the Community sample, 64% of the Clinic–No HA sample, and 75% of the Clinic–HA sample. The HHIE-total scores were used to corroborate the effects of sample type on perceived hearing difficulties reported here for the CPHI. The mean (standard error) HHIE scores for the three sample types were 12.8 (1.1), 27.2 (1.2), and 41.6 (1.5) for the Community, Clinic–No HA, and Clinic–HA samples, respectively (lower scores indicating less perceived hearing handicap). There was a significant effect of sample type on HHIE scores, *F*(2, 593) = 120.3, *p* < .001, and post hoc Bonferroni-adjusted paired comparisons revealed that all sample types differed significantly (*p* < .05) in HHIE score from one another. This was without controlling for age and better-ear PTA4, the two main covariates based the CPHI analyses, which are likely to impact HHIE scores significantly and are known from the prior analyses of CPHI scores to differ significantly across the sample types. The GLM analysis of HHIE scores was repeated with age and better-ear PTA4 as covariates. Age, *F*(1, 591) = 7.8, *p* < .01, PTA4, *F*(1, 591) = 201.4, *p* < .001, and sample type, *F*(2, 591) = 10.1, *p* < .001, all had significant effects on HHIE-total scores. Post hoc Bonferroni-adjusted paired comparisons among the three sample types found that all sample types differed significantly (*p* < .05) from one another when evaluated at a mean age of 70.4 years and a mean better-ear PTA4 of 32.2 dB HL. These findings from most participants included in the CPHI analyses support the principal findings of those CPHI analyses. First, there are significant differences in age, hearing loss, and perceived hearing difficulties across the three sample types, but the latter may be mediated to some extent by the differences across the sample types in age and hearing loss. Second, when the differences across sample types in age and hearing loss were controlled statistically, residual differences in perceived hearing difficulties remain across the three sample types.

## Discussion

### CPHI Comparisons to Other Clinical Samples

Figure 2 presented the mean CPHI scale scores and standard errors for each sample type in this study together with the means and standard deviations from the representative clinical data set of Erdman and Demorest (1998a). As noted, there was good agreement between those normative values and the two clinical sample types of this study: Clinic–No HA and Clinic–HA. Recall that almost 40% of the normative clinical sample of Erdman and Demorest (1998a) was comprised of individuals currently wearing hearing aids. We conclude that the CPHI scale scores for our two clinical samples are in good agreement with prior normative clinical data from Erdman and Demorest (1998a) for the CPHI.

Erdman and Demorest (1998b) examined associations among various participant characteristics and CPHI scale scores. Sequential regression analyses revealed that the four main predictors among those investigated were audiometric measures, with better-ear PTA4 being the strongest predictor among those measures, age, sex, and education level. All these predictors except educational level were examined in this study. Findings very similar to those reported here were obtained by Erdman and Demorest (1998b). As in this study, hearing loss was the strongest predictor for most scales, but especially for the CP scales. Thus, our clinical data compare favorably to those normative clinical data of Erdman and Demorest (1998a, 1998b) not just in mean values but in the underlying factors mediating performance on the CPHI.

Garstecki and Erler (1999) also examined the effects of sex on CPHI scale scores among a group of 301 older adults (age ≥ 65 years). These investigators observed a significant effect of sex for five of the 20 CPHI scales examined here. As in the present analyses, these investigators also controlled for differences in age and better-ear PTA4. Garstecki and Erler (1999), however, also controlled for several additional demographic variables, including income, education level, intelligence, employment, and marital status. Only two of the five significant effects of sex observed by Garstecki and Erler (1999) overlapped with those reported here: CS-nonverbal and PA-denial. Both scales showed sex differences in Erdman and Demorest (1998b) as well. In all three studies, women made greater use of nonverbal strategies to enhance communication, such as positioning oneself for best listening conditions, than men and were more likely to deny negative emotional reactions to communication difficulties than men.

All told, the clinical data from the present analyses of CPHI scale scores from older adults are consistent with the prior data from large-scale studies of clinical samples (Erdman & Demorest, 1998a, 1998b; Garstecki & Erler, 1999). These prior studies, however, did not partition their clinical samples into adherents versus nonadherents as was done here. Moreover, the prior literature included only clinic samples and not broader community samples in the compilations of CPHI scale scores. The differences across these three sample types were the primary focus of this study.

### Comparison of the Community Sample to the Two Clinical Samples

Without controlling for age, hearing loss (PTA4), or sex differences across the three sample types, significant effects of sample type were observed for 19 of the 20 CPHI scales; all except CE-communication need. Subsequent post hoc paired comparisons among the three sample types for these 19 CPHI scales revealed that all three sample types differed significantly most often (15 of 19 CPHI scales) with the Community sample differing from both of the clinic samples but the two clinic samples not differing significantly from one another. Recall that the Community sample was younger and had lower better-ear PTA4 values than the two clinic samples whereas the two clinic samples differed only in PTA4 with the hearing-aid adopters having more audiometric hearing loss. This suggests, not surprisingly, that an older adult with more hearing loss and a more advanced age is more likely to have an audiologic evaluation, and further increases in hearing loss increase the likelihood of adhering to recommendations to acquiring hearing aids.

A more interesting question, however, is whether differences in older adults' perceived communication difficulties and their reactions to those difficulties among the sample types remain after controlling for hearing loss and age. This was addressed here by a series of GLM analyses examining the effects of sex and sample type while statistically controlling for age and better-ear hearing loss (PTA4). Here, the Community sample still consistently differed significantly from the two clinic samples. In particular, even when controlling for the significant differences between sample types in age and hearing loss, the Community sample reported better communication performance in all settings and had less awareness of communication problems relative to one or both clinical samples (see Figure 4 and bottom of Figure 3). The lower CP-problem awareness score for the Community sample is coupled with a lower PA-denial score, which also indicates those in the Community sample more frequently denied difficulties and the emotional reactions to them. Three of the four scales examining the CE scale showed significant differences between the Community sample and one or both clinical samples. CE-communication need scores were highest for the Community sample indicating greater demands placed on the individual for communication compared to the clinic samples. The higher scores on CE-physical characteristics scale for the Community sample indicate that the environment, including background noise, soft speakers, and so forth, was judged to be less difficult by them compared to the clinical samples. The Community sample also felt that they less frequently encountered negative attitudes from their communication partners (CE-attitudes of others) compared to the clinic samples. Those in the Community sample also made less frequent use of verbal and nonverbal strategies to enhance communication (CS-verbal, CS-nonverbal) compared to one or both clinical samples. This might be due, in part, to not having visited an audiology clinic for evaluation, receiving counseling and education about communication strategies. Finally, the Community sample had fewer problems with their personal adjustment to hearing loss than one or both clinical samples for five of the nine CPHI-PA scales, including a higher general self-acceptance, less exaggeration of one's responsibility for successful communication, less discouragement from continued frustrations with communication, less stress resulting from communication difficulties, and less withdrawal from social interactions in difficult communication situations. As noted earlier, however, those older adults in the Community sample also had greater denial of communication difficulties than one or both clinical samples.

As noted, the initial GLM analysis that did not control for age or hearing loss found that all three sample types differed on 15 of the 20 CPHI scales examined here and that the Community sample differed from both clinic samples for four of the remaining five scales. Even after controlling for hearing loss and age differences among the three sample types*,* the Community sample differed from one or both clinical samples on 15 of 20 scales (see bottom of Figure 3 and Figure 4). In general, older adults in the Community sample perceived few communication performance problems and, consequently, made less use of compensatory strategies and had better personal adjustment to communication difficulties. The lower performance of the Community sample on the CP-problem awareness and PA-denial scales, however, suggests that this overall pattern of denial may underlie the differences on those other scales leading to a lower likelihood of such individuals reporting to the audiology clinic for evaluation of their communication difficulties and perhaps a lower likelihood of adhering to recommendations for acquiring hearing aids. Given the well-known impact of hearing loss on significant others of the older person with hearing loss (Preminger et al., 2015; Scarinci et al., 2008, 2012), it would have been ideal to have also included a measure of hearing difficulties as observed by the significant other. Doing so may have provided even stronger evidence that the responses of those in the Community sample, with hearing loss similar to that of the clinic samples, resulted primarily from denial or a lack of awareness of such difficulties. Better delineation of the underlying reasons for the failure of older adults in the community with significant hearing loss to seek help at the clinic or elsewhere may also lead to better approaches to intervention for those same individuals. Social support and self-efficacy, for example, are two approaches to intervention that may be even more critical for older adults in denial of communication difficulties (e.g., Pichora-Fuller et al., 2016).

A key assumption here is that the Community sample from the MUSC data set is representative of a broader community sample, rather than a clinical sample. To evaluate this in more detail, basic demographic and audiologic data are compared between the MUSC Community sample used here and the population study of the community in Beaver Dam, Wisconsin, referred to as the Epidemiology of Hearing Loss Study (EHLS; Cruickshanks et al., 1998). In both samples, those cases who reported hearing aid use were deleted. The second wave of the EHLS longitudinal study, EHLS2, included 2,681 individuals ranging in age from 52.8 to 97.0 years of age. The Community sample used here was comprised of data from 243 individuals ranging in age from 50 to 88 years of age. In both samples, 58% of the participants were female. Table 2 compares the two samples in more detail for a series of measures stratified by the three most prevalent World Health Organization hearing-impairment (WHO-HI) grades (Stevens et al., 2013): (a) normal, better-ear PTA4 ≤ 20.5 dB HL; (b) mild, 20.51 ≤ better-ear PTA4 ≤ 35.5 dB HL; and (c) moderate, 35.51 ≤ better-ear PTA4 ≤ 50.5 dB HL. These three WHO-HI grades constitute 99.2% of the MUSC Community sample and 93.4% of the EHLS2 sample and the relative distribution of WHO-HI grades across these three most prevalent grades is very similar in both samples. The means and standard deviations for the better-ear PTA4 values and ages across the two samples are also very similar for each WHO-HI grade. Finally, scores were available from both data sets for the HHIE Screener (HHIE-S; Ventry & Weinstein, 1983; Weinstein, 1986). Scores on the HHIE-S range from 0 to 40 with higher numbers representing greater perceived hearing difficulties and emotional reactions to them (opposite to the CPHI). The means and standard deviations for the self-reported HHIE-S scores are again similar for the MUSC Community sample and the EHLS2 samples within each WHO-HI grade, although the MUSC sample consistently exceeds the EHLS2 sample by a few percentage points. It is concluded that the Community sample included in these data analyses is representative of other similar and broader samples of older adults in the community.

**Table 2.** Comparison of Community sample type (MUSC, *n* = 241) to a population sample, the Epidemiology of Hearing Loss Study (EHLS, *N* = 2,681).

**WHO-new HI grade**	**%MUSC**	**%EHLS**	***M* (*SD*)** **PTA4 BtrE** * **MUSC**	***M* (*SD*)** **PTA4 BtrE** * **EHLS**	***M* (SD)** **HHIE-S** **MUSC**	***M* (SD)** **HHIE-S** **EHLS**	***M* (*SD*)** **Age (yrs)** **MUSC**	***M* (*SD*)** **Age (yrs)** **EHLS**
Normal	53.1	48.7	11.9 (5.6)	11.7 (5.4)	4.0 (5.0)	1.9 (3.9)	67.3 (5.6)	64.2 (7.3)
Mild	35.4	29.8	27.9 (3.9)	27.6 (4.3)	9.7 (9.0)	5.8 (7.6)	68.9 (5.4)	70.9 (8.7)
Moderate	10.7	14.9	42.0 (4.1)	41.3 (4.1)	16.5 (7.9)	11.3 (9.9)	71.0 (6.8)	76.5 (8.6)

* In both samples, data from those using hearing aids were excluded. For each sample, the data are shown stratified by World Health Organization (WHO) new hearing impairment (HI) grade (Stevens et al., 2013). Although the *n* for the MUSC Community sample was 243, two individuals had hearing loss classified beyond the “Moderate” category and were not tabulated here. MUSC = Medical University of South Carolina; PTA4 = four-frequency pure-tone average; BtrE = better ear; HHIE-S = Hearing Handicap Inventory for the Elderly scores; HI = hearing impairment; values are in dB HL.

### Comparisons Between the Two Clinical Samples

As noted in the results, another set of GLM analyses looked more closely for differences between the two clinical samples after controlling for age and hearing loss (see Figure 5). Significant differences between the Clinic–No HA and Clinic–HA samples were observed on seven of the 20 CPHI scales. The pattern that emerged was that adherents and nonadherents differed, with adherents (a) perceiving greater communication difficulties in all situations, (b) having better awareness of communication problems, (c) making more frequent use of nonverbal strategies to enhance communication, (d) being more accepting of hearing loss, and (e) being more likely to place more responsibility for communication difficulties on others. Again, these differences in CPHI scale scores cannot be attributed to differences in either age or hearing loss as these served as covariates. Rather, the observed differences capture residual perceived differences in communication difficulties and reactions to those difficulties between the two clinical samples after adjusting for differences in age and hearing loss.

As noted in the introduction, there have been a couple of studies examining differences in CPHI scale scores for clinical samples of adherents (who followed the clinician's recommendation for hearing-aid uptake) and nonadherents (who did not). Garstecki and Erler (1998) observed differences, when analyzed separately for each sex, on only two of the 20 CPHI scale examined here: CS-verbal strategies (for females) and PA-accept loss (for males). The latter was one of the seven differences in CPHI scale scores between the two clinical samples observed here (see Figure 5). There were about 35 participants in each sex/hearing-aid group in Garstecki and Erler (1998). In addition, Humes et al. (2003) matched three groups for age, sex, and hearing loss, with about 25 participants per group, prior to examining differences in CPHI scale scores. The only significant differences observed in that study were between the nonadherents and the other two clinical groups, both of whom took up hearing aids with one keeping them (at least until the 6-month follow-up) and the other returning them. The latter two groups would be akin to the Clinic–HA sample whereas the first group, nonadherents, is equivalent to the Clinic–No HA sample in this study. Humes et al. (2003) observed significant differences between the nonadherents and the other two groups on several CPHI scales (CP-problem awareness, PA-self accept, PA-exaggerated responsibility, PA-stress, and PA-denial scales). The only overlap with the current data is for the CP-problem awareness scale. The direction of the difference on this CPHI scale is the same in both studies with nonadherents (Clinic–No HA) having significantly lower awareness of their communication problems than adherents (Clinic–HA). The pattern of findings cross CPHI scales in Humes et al. (2003) is more like the pattern observed in the bottom panel of Figure 4 between the Community and the Clinic–No HA samples. This study had much larger sample sizes for the two clinical groups than in either Garstecki and Erler (1998) or Humes et al. (2003) and, therefore, we have greater confidence in the pattern of differences between adherents and nonadherents in Figure 5 than in the earlier smaller sample studies. An important assumption here, however, is that all of those in the Clinic–No HA sample were, in fact, counseled to acquire hearing aids. If the recommendation to acquire hearing aids was never made to those in the Clinic–No HA sample, they could not be considered “nonadherents.” There is no way to discern what percentage in each of those samples received this recommendation. Based on data from 1,382 older adults screened for hearing loss by Yueh et al. (2010) and then subsequently followed over the next year, about two thirds of those who were evaluated by an audiologist following screening failure received recommendations to acquire hearing aids. It is assumed here that the vast majority of those in the Clinic–No HA sample had, in fact, been advised to acquire hearing aids and chose not to do so. Of course, because audiologists routinely rely heavily on audiometric data to make recommendations for amplification, the likelihood of making such a recommendation would vary with the severity of hearing loss.

The results obtained for the HHIE for most participants included in the CPHI analyses support the principal findings on the impact of sample type on the CPHI. Humes et al. (2003), using a matched-group approach with matching for age, hearing loss, and sex, also found significant differences (*p* < .05) in HHIE total scores between groups akin to the Clinic–No HA and Clinic–HA sample types investigated here.

### Study Limitations

The greatest limitation to the study relates to the way in which membership in one of the three samples was determined. For the Community sample, for instance, it is known that the members of that sample, (a) volunteered for the MUSC longitudinal study of hearing, (b) were not referred to the study from the MUSC audiology clinic, and (c) were not current hearing-aid users. As noted previously, this does not preclude them from having had a hearing evaluation previously but at a non-MUSC clinic or from having tried hearing aids previously but discontinued their use prior to enrollment in the longitudinal study. Likewise, for the Clinic–No HA sample, it is known that they received an audiological evaluation at either the MUSC or the IU clinic, but for the MUSC members of that sample, it is only known that they were not currently using hearing aids. That is, it is possible that they had tried hearing aids (adherents) but stopped using them prior to enrollment in the MUSC longitudinal study. Whereas, the MUSC study confirmed current hearing aid usage, inquiries about prior hearing aid usage were not made. For the members of the Clinic–No HA sample from IU, those who had never tried hearing aids comprised the presumed group of nonadherents. Furthermore, for the entire Clinic–No HA sample, it is presumed that hearing aids were recommended for every member of this sample, but they declined to pursue them, thereby making them nonadherents. For the Clinic–HA sample, considered to be adherents, it is again assumed that they adhered to the recommendation which was to acquire hearing aids. It is, of course, unlikely that hearing aids were acquired without a clinical evaluation and recommendation. Nonetheless, because this was not asked directly of each participant at the time of enrollment in either the MUSC or the IU studies, this cannot be confirmed directly.

In addition to this main limitation, there are other common limitations on the generalization of these findings. For example, older adults from only two communities, Charleston, SC and Bloomington, IN, participated and the participants were overwhelming White and non-Hispanic. This limits the broad generalization of the findings to other demographic groups. In addition, all members of all three samples were research volunteers and those who volunteer for research studies may not be representative of the broader community from which they were drawn.

## Conclusions

In conclusion, this study found significant differences in CPHI scale scores (and HHIE scores) across Community, Clinic–No HA, and Clinic–HA sample types. Hearing loss, and to a lesser extent, age and sex, impacted the effects of sample type on these self-report measures. It is not surprising that an individual's likelihood of taking action, whether to get a hearing evaluation or to acquire hearing aids after such an evaluation, increases as with age and the severity of hearing loss. Importantly, even when controlling for hearing loss and age, many significant differences in CPHI scale scores (and HHIE scores) remained across these sample types. Those who sought clinical evaluation of their hearing loss, regardless of hearing-aid decision, differed from those in the community who did not. The major differences were in the degree of perceived communication difficulty, with those not seeking clinical evaluation reporting less perceived difficulty, and a greater denial or lack of awareness of communication problems among those in the community who did not seek clinical assistance. For the two groups who did seek clinical evaluation of their hearing difficulties, one obtaining hearing aids and the other not doing so, those who acquired hearing aids perceived greater difficulty in communicating in all environments, had greater awareness of communication difficulties, were more likely to use nonverbal strategies to compensate for their difficulties, were more accepting of their hearing loss, but tended to allocate more responsibility for difficulties to their communication partners. Thus, beyond the differences in measured audiometric hearing loss and age across the three sample types examined here, there were real differences in their perceived hearing difficulties and their reactions to them. This reinforces the notion that, ultimately, it is the older adult's self-awareness of hearing difficulties that is most critical to seeking help for hearing problems, as well as remediation for those problems. Efforts to promote and make available self-assessment of hearing difficulties by older adults should be advocated and included in opportunities for hearing screening. In that regard, Humes (2021) has recently proposed the development of an approach to auditory wellness centered on the use of the HHIE. As shown here, the HHIE also reflected differences among the three sample types studied here. In addition, the HHIE is much more practical to self-administer or to administer in a clinical setting than the 145-item CPHI. Cassarly et al. (2020) have recently shortened the 25-item HHIE down to an 18-item self-report measure with sounder psychometric properties than the original.
